# Ocular and orbital manifestations in VEXAS syndrome

**DOI:** 10.1038/s41433-024-03014-3

**Published:** 2024-03-28

**Authors:** Muhammad Abumanhal, Igal Leibovitch, Michael Zisapel, Tali Eviatar, Yonatan Edel, Ran Ben Cnaan

**Affiliations:** 1 Oculoplastic, Orbital and Lacrimal Institute, Division of Ophthalmology, Tel Aviv Medical Center, Tel Aviv, Israel; 2 Sackler School of Medicine, Tel Aviv University, Tel Aviv, Israel; 3 Rheumatology Department, Tel Aviv Medical Center, Tel Aviv, Israel; 4 Department of Internal Medicine ‘B’, Assuta Ashdod University Hospital, Ashdod, Israel; 5 Faculty of Health Science, Ben Gurion University of the Negev, Beer-Sheva, Israel

**Keywords:** Eye manifestations, Inflammatory diseases

## Abstract

**Background:**

VEXAS (Vacuoles, E1 enzyme, X-linked, Autoinflammatory, Somatic) is a hematoinflammatory disease that typically affects adults. It results from a somatic mutation of the E1 ubiquitin conjugating enzyme encoded by the UBA1 gene. VEXAS is frequently accompanied by myelodysplastic syndrome (MDS). The purpose of this study is to describe the ocular and orbital manifestations of VEXAS patients in a case series in our medical centre.

**Methods:**

A retrospective chart review was performed for all patients who were diagnosed with VEXAS syndrome in a tertiary medical centre over two years.

**Results:**

Eight patients were identified with VEXAS. In six patients, the diagnosis was confirmed by genomic sequencing. Two patients were identified based on their phenotype. All patients were males. The mean age at diagnosis was 78.7 years. In two patients, the ocular manifestation was the presenting symptom for VEXAS. Seven patients (87.5%) had history of MDS. Systemic inflammation manifestations include: skin rash (*n* = 5), recurrent fevers (*n* = 2), relapsing polychondritis (*n* = 2), pleuritis and pleural effusion (*n* = 2), poly arteritis nodosa- PAN (*n* = 1) and thrombophlebitis (*n* = 1). Seven (87%) patients were presented with periorbital oedema. Three patients showed orbital inflammation. Dacryoadenitis was observed in two patients, and extraocular muscle (EOM) myositis was detected in two patients. Four patients demonstrated ocular inflammation such as: episcleritis, scleritis and anterior uveitis.

**Conclusion:**

ocular manifestations in VEXAS include orbital inflammation, dacryoadenitis, myositis, uveitis, scleritis, episcleritis and periorbital oedema. We recommend that in old male patients, with history of haematological disorder, presenting with ocular symptom, VEXAS investigation should be taken into consideration.

## Introduction

VEXAS syndrome was first introduced by Beck et al in October 2020 [1]. It is an adult-onset autoinflammatory syndrome that primarily affects older males. VEXAS (V- vacuoles, E- E1 enzyme, X- X-linked, A- autoinflammatory, S- somatic) results from somatic mutation of the E1 ubiquitin-conjugating enzyme encoded by the UBA1 gene. Common systemic features include recurrent fever, ear and nose chondritis, skin lesions, venous thromboembolism, and symptomatic and asymptomatic pulmonary pathologies (pulmonary infiltrates and pleural effusion). Haematologic abnormalities consisting of macrocytic anaemia and sometimes other cytopenias are also common, frequently accompanied by myelodysplasia or frank myelodysplastic syndrome (MDS) [1]. The affected bone marrow is characterised by the presence of myeloid vacuoles [1]. Patients with VEXAS were found to have overexpression of inflammatory cytokines, interferon-gamma, interleukin-8, and interferon-inducible protein 10. As a result, these proteins and inflammatory mediators accumulate and activate innate immune pathways that cause multisystem inflammation with multi-organ involvement including skin, cartilages, eyes, lungs, blood vessels, and joints [1]. However, the exact mechanism underlying the relapsing inflammatory symptoms remains poorly understood.

Currently, there is no standardised treatment for VEXAS. However, most patients require moderate to high doses of corticosteroids and other immunosuppressive agents to control inflammatory symptoms [2, 3]. In refractory cases, tocilizumab (anti-IL-6), cyclosporine, JAK inhibitors, and even hematopoietic stem cell transplantation have been introduced as treatment options [4]. Severe skin reactions to anakinra (interleukin-1 receptor antagonist) were reported in 62% of patients [1]. It is characterised by a delayed injection site reaction with erythematous infiltrated plaques.

Beck et al. reported periorbital inflammation in some VEXAS patients (28%) [1]. However, since their description in 2020, the number of patients diagnosed with VEXAS has rapidly increased, and the spectrum of clinical manifestation has expanded to include various ocular manifestations. These include orbital inflammation [5–9], orbital myositis [10], dacryoadenitis [11], cellulitis [12, 13], ophthalmoplegia caused by polycranial neuritis [14], optic neuritis [7], scleritis [5, 9, 15, 16], uveitis [15, 16], ocular palsy [9], and retinal vasculitis [16].

The aim of this study is to describe the ocular and orbital manifestations of VEXAS syndrome in patients treated or consulted by a tertiary medical centre. To our knowledge, this is the first and largest series discussing this topic.

## Materials and methods

A retrospective chart review was conducted for patients who were genetically or phenotypically diagnosed with VEXAS syndrome and were treated or consulted by physicians in a single tertiary medical centre from January 2021 to December 2022. The study included patients who were diagnosed with a reported mutation in the UBA1 gene, confirmed by genomic sequencing.

Additionally, a chart review was performed for deceased patients with haematological disorders (MDS, cytopenia, macrocytic anaemia) and systemic inflammatory diseases (vasculitis, polyarteritis nodosa-PAN, chondritis, skin rash, and recurrent fevers) who underwent bone marrow aspiration. Bone marrow revision was performed to identify vacuoles. Two patients with vacuoles and clinical VEXAS features were identified and considered as presumed VEXAS subjects.

Electronic chart review evaluated ophthalmology visits in the emergency department and clinic, as well as hospitalisations in the ophthalmology department. Data collection included demographic characteristics (sex, age at diagnosis and at first ocular manifestation), past medical and ocular history, visual acuity, ocular examination, type of ocular manifestation, laterality, recurrence, haematologic disorder, systemic inflammatory manifestation, immunologic serologies, orbital pathology results, treatment, and duration of follow-up. If performed, imaging studies were reviewed by an ophthalmologist and a radiologist. Treatments such as corticosteroids, systemic immunosuppressive medications, and biologic agents were recorded.

The UBA1 mutation was determined by Sanger sequencing of the UBA1 gene from bone marrow or blood samples. The study was conducted in accordance with the Declaration of Helsinki, and ethical approval was obtained from the institutional review board. All data were recorded in Microsoft Excel (2020)™ and analysed using SPSS version 23 (SPSS Inc., Chicago, IL, US).

## Results

Eight patients were diagnosed with VEXAS syndrome. Six of them were confirmed by genomic sequencing. Four patients (66%) carried the c.121A>G, Met41Val mutation, one (16%) patient had the c.121A>C, Met41Leu mutation, and one (16%) had a splice acceptor site mutation. Two patients who were already deceased before VEXAS syndrome was described were identified based on their phenotype and the presence of vacuoles on their bone marrow aspiration.

**Fig. 1:**
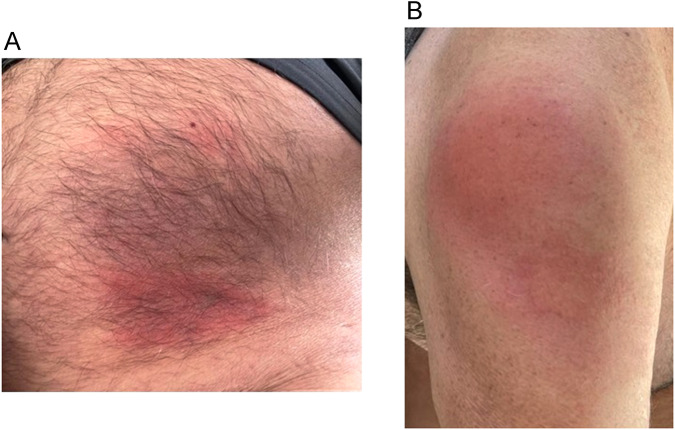
Skin reaction to anakinra injection in VEXAS patients. Case 4 (**A**) & 5 (**B**) with severe skin drug reaction to anakinra.

Table 1 shows the demographic characteristics and systemic manifestations of the patients. All patients were male, with a mean age of 78.7 years ±5.3 years at diagnosis and 76.2 ± 5.9 years at first ocular presentation. In two patients (25%), ocular manifestations were the presenting symptoms of VEXAS. Seven patients (87.5%) had a history of MDS, while one patient (12.5%) suffered from macrocytic anaemia. Systemic inflammatory manifestations included recurrent fevers (*n* = 7, 87.5%), neutrophilic dermatosis and/or vasculitic rashes (*n* = 7, 87.5%), recurrent ear chondritis (*n* = 1, 12.5%), pulmonary ground glass opacities (GGO) and/or pleural effusion (*n* = 6, 75%), and thrombophlebitis (*n* = 2, 25%). One patient (case 1) did not show any systemic clinical features, and the ocular symptoms were the only non-haematologic manifestation. Three patients (37.5%) experienced injection site cutaneous drug reactions to Anakinra [Fig. 1]. To date, three patients (37.5%) have passed away- two before the actual VEXAS diagnosis, and one about two years after being diagnosed.

**Table 1:** Summary of the demographic characteristics and systemic features of all cases.

Case	Gender	Age at diagnosis	Haematologic disorder	Mutation	Systemic inflammation features & survival	Treatment
1	M	87	Macrocytic anaemia	Valine	None Alive	GCS
2	M	80	MDS	Splice Acceptor Site	Recurrent fever, SSS, GGOs and pleural effusion, laryngitis, sinus vein thrombosis Deceased	GCS, TOC
3	M	74	MDS	Leucine	Recurrent fever. SSS, aortitis on CT, cutaneous vasculitis, severe drug reaction to anakinra, GGOs and pleural effusion Alive	GCS, HCQ, MTX, COL, DAP, ANA
4	M	75	MDS	Valine	Recurrent fever, SSS, severe drug reaction to anakinra, DVT, superficial thrombophlebtits, asymptomatic GGOs and pleural effusion Alive	GCS, TOC, COL
5	M	75	MDS	Valine	Recurrent fever, SSS, severe drug reaction to anakinra, PAN like syndrome, superficial thrombophlebtits, GGOs and pleural effusion Deceased	GCS, MTX, CsA, CYC, ANA, TOC, CAN
6	M	76	MDS	Valine	Recurrent fever, SSS, myositis, severe symptomatic GGO’s (respiratory failure) Alive	GCS, TOC
7^a^	M	75	MDS	NA	Recurrent fever, SSS, DVT, superficial thrombophlebitis, pericarditis, symptomatic GGOs Deceased	GCS
8^a^	M	88	MDS	NA	Recurrent fever, SSS, recurrent ear chondritis, motor neuropathy Deceased	GCS, MTX

*M* male, *MDS* myelodysplastic disorder, *PAN* polyarteritis nodosa, *SSS* skin sweet syndrome, *GGO* pulmonary ground glass opacity, *DVT* deep vein thrombosis, *GCS* glucocorticosteroids, *TOC* tocilizumab, *HCQ* hydroxychloroquine, *MTX* methotrexate, *COL* colchicine, *DAP* dapsone, *ANA* anakinra, *CsA* cyclosporine A, *CYC* cyclophosphamide, *CAN* canakinumab.

^a^Case 7 and 8 considered presumed VEXAS. Genomic sequencing was not performed and post-mortem revision of the bone marrow samples demonstrated vacuoles.

Table 2 presents ocular presentations of the patients. Seven patients (87.5%) had periorbital oedema. Three patients underwent computed tomography (CT) which showed orbital inflammation. One demonstrated dacryoadenitis, the second had extraocular muscle (EOM) myositis, and the third was diagnosed with both dacryoadenitis and myositis. Five patients (62.5%) demonstrated ocular inflammation such as episcleritis, posterior scleritis, and mild anterior uveitis. Four patients (50%) had bilateral eye involvement; however, none of them presented with both eyes simultaneously. The clinical course was relapsing-remitting with recurrent episodes in six patients (75%).

**Table 2:** Summary of the ocular manifestations and clinical features.

Case	Age at 1^st^ ocular manifestation	Ocular presentation	Side	Episode	Imaging
1	87	PE, OIS, dacryoadenitis	Bilateral	Recurrent	CT
2	75	PE, OIS, dacryoadenits, myositis, post scleritis	Bilateral	Recurrent	CT, US
3	72	PE, episcleritis, uveitis	Unilateral	Recurrent	UBM
4	73	PE	Bilateral	Recurrent	None
5	69	Post scleritis, ant uveitis	Unilateral	Recurrent	US
6	75	PE	Unilateral	Single	None
7	74	PE, OIS, myositis, post scleritis	Bilateral	Recurrent	CT
8	85	PE, ant uveitis	Unilateral	Single	None

*PE* periorbital oedema, *OIS* orbital inflammatory syndrome, *Abx* antibiotics.

### Case 1

**Fig. 2:**
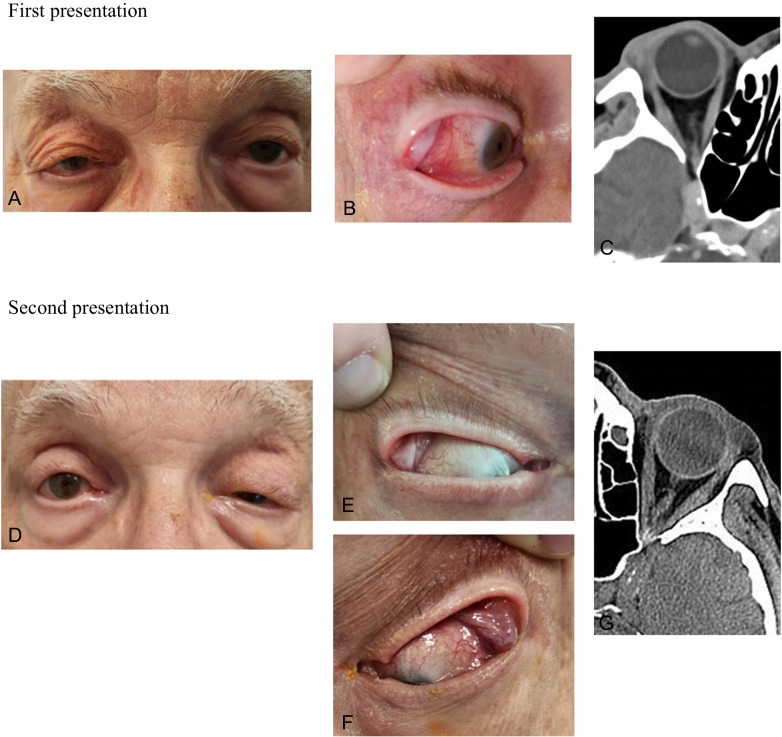
case number 1. First presentation (November 2022) of the right eye shows periorbital oedema (**A**), lacrimal gland swelling and temporal conjunctival injection (**B**). CT demonstrated lacrimal gland enlargement and enhancement of distal part of the lateral rectus at the insertion site (**C**). Second presentation (December 2022) of the left eye illustrates periorbital oedema and ptosis (**D**), resolving of right lacrimal gland swelling (**E**) and left eye dacryoadenitis (**F**, **G**).

An 87-year-old male with a history of macrocytic anaemia presented to our department in November 2022 with mild periorbital swelling and tenderness on the right side that had lasted for one week. Ocular examination revealed diplopia, limitation in the right eye abduction, mild conjunctival chemosis, and lacrimal gland enlargement [Fig. 2]. CT showed periorbital oedema, lacrimal gland swelling, enhancement of the distal part of the lateral rectus at the insertion site, and intraconal fat infiltration. The patient was initially treated with antibiotics for suspected infectious orbital cellulitis following a recent complicated maxillofacial procedure and had full resolution of symptoms within two weeks. However, one month later, the patient presented again with left eye dacryoadenitis and periorbital oedema resembling the features of the first presentation on the right side. Laboratory tests demonstrated pancytopenia with macrocytosis, increased CRP (41 mg/L), moderate elevated rheumatoid factor (RF) 42.6 IU/ml, elevated IgG4 (3.18 g/L), and positive thyroglobulin antibody (3155 U/L). Further immunologic investigations were negative. Sanger sequencing revealed a UBA1: c.121A>G; p. (Met41Val) variant diagnostic of VEXAS syndrome. Prednisone treatment was initiated, and significant improvement was observed within two weeks. To date, he is under treatment with 15 mg prednisone. The patient denied immunotherapy treatment.

### Case 2

**Fig. 3:**
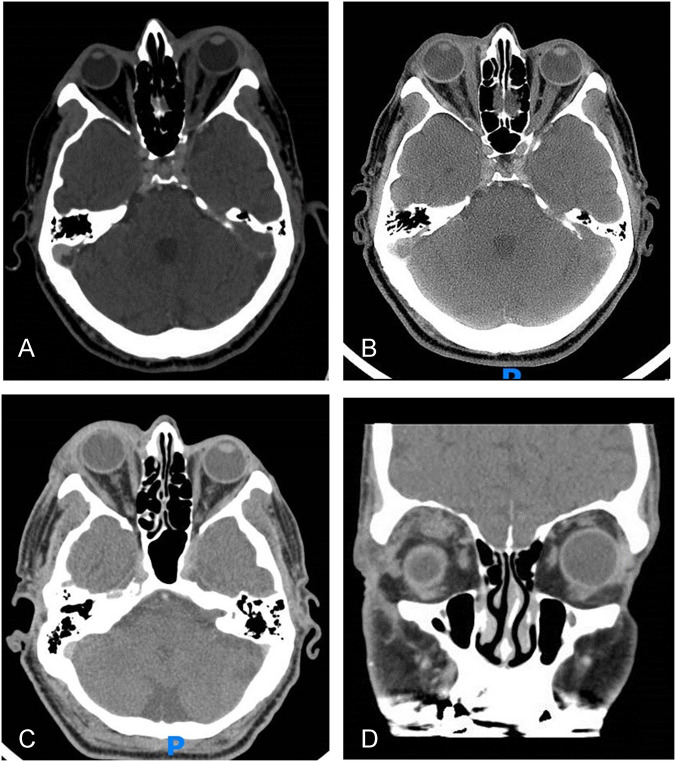
case number 2, CT scans in different presentations. **A** First presentation (March 2017) shows proptosis of the left eye, preseptal swelling, medial rectus and lacrimal gland enlargement. **B** Presentation (February 2018), right eye proptosis and medial rectus enlargement. **C**, **D** presentation (December 2018), axial scan (**C**) demonstrates right proptosis, preseptal swelling and lacrimal gland enlargement. Coronal scan (**D**) shows superior rectus swelling accompanied with infiltration the surrounding fat.

In March 2017, a 75-year-old male presented to our department with a one-week history of left eye periorbital oedema, proptosis, and chemosis [Fig. 3]. CT revealed lacrimal gland and medial rectus enlargement. Blood tests showed macrocytic anaemia and leukopenia, elevated CRP (44 mg/L), positive thyroglobulin antibody (404 U/L), and thyroid peroxidase antibody (108 U/L). IgG4 was elevated (2.5 g/L), while other immunologic serologies were negative.

Ultrasound Biomicroscopy (UBM) revealed diffuse episcleral thickening. The patient was diagnosed with orbital inflammatory syndrome and treated with prednisone 60 mg/day, which resulted in a good response and regression of signs and symptoms within 10 days. Over the next two years, he was hospitalised three times in our department with alternating unilateral inflammatory orbital flares. He was treated successfully twice with prednisone, and once the symptoms resolved without treatment. The second presentation was accompanied by right eye posterior scleritis confirmed by typical T-sign in ultrasound (US). Lacrimal gland biopsy performed in December 2018 revealed patchy lymphocytic infiltrate composed of small T and B lymphocytes. IgG4 staining was negative.

Other systemic manifestations required another three hospitalisations between 2018 and 2021 in other departments with recurrent fevers, fatigue, weight loss, pulmonary ground glass opacities (GGOs), and one episode of laryngitis. His blood test deteriorated with pancytopenia and very high CRP (up to 236 mg/L). In May 2021, he was diagnosed with VEXAS syndrome after genome sequencing revealed a mutation in the splice acceptor site. This mutation was described in 6% of the patients [17]. The patient received tocilizumab and 7 mg/day of prednisone, which controlled his condition well for 18 months. In March 2023, he presented to the emergency department with confusion, general weakness, and focal seizures. CT demonstrated intracranial sinus vein thrombosis. Despite anti-coagulation treatment, the patient deceased due stroke complications and respiratory failure.

## Discussion

VEXAS syndrome is a rare autoinflammatory disorder that is acquired in adulthood and caused by mutations in the UBA1 gene. It is triggered by acquired somatic mutations in the blood tissues, resulting in hematoinflammatory manifestations [1, 18]. In this paper, we present ocular manifestations in eight patients diagnosed with VEXAS and review the myriad of ocular features reported in the literature.

The clinical spectrum of orbital involvement in VEXAS syndrome continues to be defined as more cases are confirmed and described. A literature review carried out by Koster et al. concluded that 15% of VEXAS patients experienced orbital and/or periorbital inflammation [19]. Beecher and colleagues reported a similar rate of 13.9% [11]. The reported prevalence of ocular symptoms in patients with VEXAS is between 16–39% [1, 20, 21]. Periorbital oedema was the most frequently described ocular manifestation. Georgin et al. found that 10 out of 116 patients (8.6%) presented with periorbital oedema [21]. Ferrada and colleagues reported a higher rate of 30% in a case series of 83 patients [20]. We identified 7 out of 8 (87.5%) patients with periorbital oedema. We assume that patients with periorbital oedema might be underdiagnosed since the symptoms could be mild and sometimes are managed by general physicians or outpatient ophthalmologists. Moreover, most studies reported in the literature were conducted by internal medicine specialists, specifically rheumatologists, immunologists, dermatologists, and haematologists. To our knowledge, this case series is the first reported by ophthalmologists, focusing on ocular and orbital manifestations.

In our study, we found that four patients (50%) presented with orbital inflammatory syndrome (OIS), including dacryoadenitis, posterior scleritis, and EOM orbital myositis. Other reported orbital presentations include cellulitis [12, 13], optic perineuritis [4, 6], polycranial neuritis causing ophthalmoplegia [14], peri/intraorbital panniculitis [15], and ocular palsy [9]. Reviewing the cases of VEXAS patients reported in the literature, we found that only a small subset of patients underwent orbital imaging. This may serve as an explanation for the underdiagnosis of orbital involvement.

Georgin et al. reported that 9.5% of VEXAS patients presented with uveitis [21]. Anterior uveitis was present in two patients (25%) in our series. One patient (12.5%) presented with episcleritis. Similarly, the same rate was reported in Georgin’s paper (12.1%). Other reported intraocular manifestations included optic neuritis [7] and retinal vasculitis [16].

Orbital biopsy was performed only in case number 2. The histopathology of the lacrimal gland showed a patchy T and B lymphocytic infiltrate and negative IgG4 staining. Beecher reported lacrimal gland biopsy demonstrating weak IgG4 granular staining in mast cells and very few IgG positive plasma cells [11]. Histopathologic evaluation reported by Van der made showed adipose tissue with reactive changes and panniculitis [15]. All reported biopsies were inconclusive and showed nonspecific inflammation. Therefore, based on the literature and our series, the value of orbital biopsy is limited and does not contribute to VEXAS diagnosis.

Almost all of the known pathogenic mutations leading to VEXAS syndrome involve substitutions of Methionine-41 (p.Met41). Around half of all published cases of VEXAS have the c.122T>C, p.Met41Thr substitution, while another fifth are made up of the c.121A>G, Met41Val and c.121A>C, Met41Leu substitution [4, 12, 17]. In our series, out of six patients who underwent genetic testing, four patients (66%) were diagnosed with Met41Val mutation. This mutation was also reported in VEXAS patients with ocular involvement [5–7, 11, 22]. The fact that Met41Val mutation is not the most common one and that it is highly diagnosed in our case series and in other ocular manifestations could warrant the speculation that this mutation may be related to ocular involvement in VEXAS syndrome. Further studies are needed to confirm this hypothesis.

Two patients (case 1 & 2) demonstrated elevated thyroglobulin antibody, and one (case 2) also showed a high titre of thyroid peroxidase antibody. Both patients had euthyroid function without clinical features of hyper or hypothyroidism. Both patients did not show typical EOM enlargement on CT scan compatible with thyroid ophthalmopathy. Thyroid antibodies were not undertaken in the rest of the patients. Huang reported a rate of 16.5% among VEXAS patients who had autoimmune disorders [23]. The most common was rheumatoid arthritis followed by psoriasis, hypothyroidism, and Behcet syndrome. Reviewing the literature, there was no reported data showing the correlation between thyroid antibodies and VEXAS syndrome.

All patients in this series had a good ocular prognosis. Patients with orbital involvement showed a rapid and good response to corticosteroids. Prednisone 20–40 mg/day seems to be the most effective treatment for VEXAS-related inflammation flares, including ophthalmological manifestations [9, 19, 24]. However, Beecher reported a case of recurrent dacryoadenitis refractory to methotrexate, which was controlled upon the treatment of the JAK inhibitor tofacitinib [10].

Early diagnosis of VEXAS is of high clinical importance. The detection of multisystem life-threatening syndromes such as VEXAS can prevent unnecessary immunological, histopathological, and radiological evaluations. Administration of appropriate systemic treatment in the early stages may prevent developing systemic inflammatory manifestations. In our paper, ocular manifestation was the presenting symptom in two patients (25%). Case 1 was diagnosed in the early stages and avoided needless investigations. Case 2 presented first before VEXAS was reported. He underwent extensive laboratory and imaging investigations. Since the diagnosis of VEXAS was performed, ocular and systemic manifestations were well controlled under tocilizumab and steroids. We suggest that in older male patients with haematological cytopenia (mainly MDS and macrocytic anaemia), presenting with ocular symptoms and high inflammatory markers, a genetic test to diagnose VEXAS syndrome should be undertaken.

The limitations of this study stem from its retrospective design and small sample size. In addition, the diagnosis in two patients was based on clinical and bone marrow aspiration features without genetic testing for a UBA1 mutation.

In conclusion, we have reported the largest series to date of VEXAS patients with ocular and orbital manifestations, including orbital inflammation, dacryoadenitis, myositis, uveitis, scleritis, episcleritis, and periorbital oedema. Ophthalmologists should be aware of this entity and refer suspected patients for genetic testing, which may save unnecessary testing and long wait times until a precise diagnosis is made. Due to the recent discovery of VEXAS and the heterogeneity of clinical manifestations, larger clinical series with longer follow-up periods are required to further investigate the ocular and orbital manifestations of this new clinical entity.

## Summary

### What was known before

- VEXAS syndrome is a new hematoinflammatory entity with ocular manifestation.

### What this study adds

- Ophthalmologists should be aware of this new entity and refer suspected patients for genetic testing. Early diagnosis of VEXAS has high clinical importance. The detection of multisystem life-threatening syndrome, such VEXAS, can withhold unnecessary evaluations, and administration of appropriate systemic treatment in the early stages may prevent developing systemic inflammatory manifestations.

## Data Availability

The data that support the findings of this study are available on request from the corresponding author- MA. The data are not publicly available due to containing information and images that could compromise the privacy of the patients.
